# Migration of Bone Marrow-Derived Very Small Embryonic-Like Stem Cells toward An Injured Spinal Cord 

**DOI:** 10.22074/cellj.2016.3836

**Published:** 2016-01-17

**Authors:** Zoleikha Golipoor, Fereshteh Mehraein, Fariba Zafari, Akram Alizadeh, Shima Ababzadeh, Maryam Baazm

**Affiliations:** 1 Department of Anatomy, Faculty of Medicine, Hamadan University of Medical Sciences, Hamadan, Iran; 2 Department of Anatomy, Faculty of Medicine, Iran University of Medical Sciences, Tehran, Iran; 3 Department of Tissue Engineering, Faculty of Advanced Technologies in Medicine, Tehran University of Medical Sciences, Tehran, Iran; 4 Department of Anatomy, Faculty of Medicine, Arak University of Medical Sciences, Arak, Iran

**Keywords:** Homing, Spinal Cord Injury, Migration

## Abstract

**Objective:**

Bone marrow (BM) is one of the major hematopoietic organs in postnatal life that consists of a heterogeneous population of stem cells which have been previously described. Recently, a rare population of stem cells that are called very small embryonic-like (VSEL) stem cells has been found in the BM. These cells express several developmental markers of pluri- potent stem cells and can be mobilized into peripheral blood (PB) in response to tissue injury. In this study we have attempted to investigate the ability of these cells to migrate toward an injured spinal cord after transplantation through the tail vein in a rat model.

**Materials and Methods:**

In this experimental study, VSELs were isolated from total BM cells using a fluorescent activated cell sorting (FACS) system and sca1 and stage specific embryonic antigen (SSEA-1) antibodies. After isolation, VSELs were cultured for 7 days on C2C12 as the feeder layer. Then, VSELs were labeled with 1,1´-dioctadecyl-3,3,3´,3´- tetramethylindocarbocyanine perchlorate (DiI) and transplanted into the rat spinal cord injury (SCI) model via the tail vein. Finally, we sought to determine the presence of VSELs in the lesion site.

**Results:**

We isolated a high number of VSELs from the BM. After cultivation, the VSELs colonies were positive for SSEA-1, Oct4 and Sca1. At one month after transplantation, real-time polymerase chain reaction analysis confirmed a significantly increased expres- sion level of Oct4 and SSEA-1 positive cells at the injury site.

**Conclusion:**

VSELs have the capability to migrate and localize in an injured spinal cord after transplantation.

## Introduction

Spinal cord injury (SCI) is a neurological condition that results in disability and dysfunction, inducing inflammatory responses throughout the injuredarea. This response acts not only on the inflammatory cells that migrate toward the damaged area, but also on increases the plasma chemokine levels. In response to these chemotactic factors, circulating cells such as granulocytes, lymphocytes, and monocytes increase in peripheral blood (PB) (1,2). Previous studies have shown that in addition to hematopoietic stem cells (HSCs), bone marrow (BM) contains a population of non-HSCs that can be mobilized into PB after myocardial infarctions (MI) (3) and strokes (4). These cells, very small embryonic-like (VSEL) stem cells, (5) are characterized by their very small size, large nuclei surrounded by a narrow rim of cytoplasm, and open-type euchromatin (6). VSEL stem cells express markers found in pluripotent stem cells such as stage specific embryonic antigen (SSEA), Oct-4, and Nanog, in addition to ahigh telomerase activity and capability to differentiate into cells from all three germlayers (7). Interestingly, these cells express primordial germ cell features such as C-X-C chemokine receptor type 4 (CXCR4), Stella and Fragilis. VSEL stem cells appear to be the progeny of epiblast cells (5). 

In addition to the BM, VSEL stem cells are present in cord blood (8), the heart, and brain. Probably this population of cells are deposited during early organogenesis in developing organs and play an important role in postnatal tissue turnover and regeneration (9). 

Recently, a number of studies have focused on nonHSCs for the regeneration of damaged tissue. 

In a mouse model of MI, intramyocardial injection of VSELs showed promising effects. VSELs could improve myocardial contractility and were more effective than HSCs (10). Their small size might enable their use as a unique source for cell therapy in neurological disorders such as strokes (11). 

Since CXCR4 is expressed on VSELs, these cells could migrate to the stromal derived factor-1 (SDF-1) gradient. SDF-1 is one of the most important chemokines that upregulates in damaged tissue. This chemokine regulates the trafficking of CXCR4 positive cells to tissue affected by inflammation. The SDF-1/CXCR4 axis plays pivotal roles in mobilization of progenitor stem cells during embryogenesis and tissue regeneration (12). 

This study used VSELs to regenerate a SCI from a rat model. We transplanted VSEL stem cells by intravenous injection into this rat model of SCI in order to determine if these transplanted cells could migrate into the lesion site. 

## Materials and Methods

This experimental study was conducted according to the Guidelines of Iran University of Medical Sciences for Animal Care and approved by the Ethics Committeeat Iran University of Medical Sciences. 

### Bone marrow cells

BM cells were collected from the femur and tibia of 2-4 week-old male National Medical Research Institute (NMRI) mice (Iran University, Iran) by vigorous flushing with Dulbecco’s modified Eagle’s medium (DMEM, Invitrogen, USA). Erythrocytes were removed with erythrocyte lysis buffer (Dako, USA). 

### Bone marrow-derived cell sorting

Sca^+^ and SSEA-1^+^ cells were isolated from a suspension of murine BM by multiparameter fluorescence activated cell sorting (FACS, Aria II-BD, Becton Dickinson, USA) according to standard procedures. Briefly, BM cells were resuspended in phosphate buffered saline (PBS, Invitrogen, USA) with 2% heat-inactivated fetal bovine serum (FBS, Gibco, USA). The following antibodies were used to stain these cells: fluoresceinisothiocyanate (FITC)- anti-Sca1 (Ly6A/E, 1:100, Abcam, USA) and anti- SSEA-1(1:100, Abcam, USA). Cells were incubated on ice for 30-45 minutes and washed twice, after whichgoat anti-mouse labeled with phycoerythrin (PE, 1:100, Abcam, USA) was added for 1 hour as the secondary antibody for SSEA-1. Finally, the cells were washed and resuspended for sorting in PBS/FBS at a concentration of 5-10×10^6^ cells/ml.

### Cell culture and expansion

Freshly sorted VSELs were cultured over C2C12 murine myoblast cell feeder layer that inactivated with mitomycin C in DMEM/F12 (Invitrogen, USA) supplemented with 20% FBS, 1X non-essential amino acids (NEAA, Invitrogen, USA), 50 mMbeta2mercaptoethanol (2ME, Invitrogen, USA), 2 mM Lglutamine (Invitrogen, USA) and 10^3^ U/mll eukemiainhibitory factor (LIF, Sigma, Germany) at 37˚C in humidified atmosphere of 5% CO_2_ in air. VSELs were expanded for 7 days with a change of medium every 3-4 days. 

### Immunocytochemistry

The cells were fixed with 4% paraformaldehyde (Sigma, Germany), and then permeabilized by 0.1% Triton X-100 (Sigma, Germany). Nonspecific binding was blocked by goat serum (Sigma, Germany). The following combinations of primary and secondary antibodies were used for double staining: FITC anti-Sca1, anti-SSEA-1, and goat anti-mouse labeled with PE (1:100, Abcam, USA, vs. anti-SSEA-1). Cells were incubated overnight with primary antibody at 4˚C. After washing, cells were incubated with secondary antibodies for 1 hour at room temperature. The nuclei were counterstained with Hoechst stain (Sigma, Germany). 

### Animals

The animals, 12-week-old male Wistar rats (250300 g) were maintained in a pathogen-free and climate-controlled environment with free access to water and food. Rats were kept at a temperature of 20˚C and exposed to alternate 12 hour light and dark cycles. We randomly divided the animals into the following 4 groups (n=5 per group) prior to the surgical procedure: SCI with VSEL treatment (experimental group), SCI with C2C12 treatment (vehicle group), SCI without any treatment (sham group), and normal animals with no SCI that received VSEL treatment (control group). 

### Spinal cord injury procedure

Each ratwas deeply anesthetized via an intraperitoneal injection of ketamine and xylazine (80.5 mg/kg, Razi Co., Iran). The animals were placed in the prone position and surgery was performed under sterile conditions. The surgical area was shaved and disinfected with a povidone iodine solution. A skin incision and blunt dissection of the muscle layers over the area were performed. The clip compression injury was performed at the region of the 9^th^-10^th^ thoracic segment by removal of the dorsal processes of the 9^th^ and 10^th^ thoracic vertebrae. The spinal cord was compressed for 1 minute dorsoventrally (13,14). The animals received postoperative care that included Ringer’s lactate solution (1 ml, subcutaneously) for 7 days and penicillin/streptomycin (intramuscularly, Invitrogen, USA) for 3 days. Additionally, the rats’bladders were manually expressedtwice per day until the return of the bladder reflex. 

### Transplantation

VSELs were labeled with the fluorescent lipophilic tracer 1,1´-dioctadecyl-3,3,3´,3´-tetramethylindocarbocyanine perchlorate (DiI, Invitrogen, USA) according to the manufacturer’s protocol. Briefly, cells were incubated in 1 μg/ mL DiI solution for 20 minutes at 37˚C. After incubation, cells were washed and resuspended in PBS. At 7 days after the SCI, 1×10 ^6^cells/200 μl were injected intravenously through the tail vein. All animals were sacrificed 28 days after treatment. 

### Tissue preparation and immunohistochemistry

Rats were deeply anesthetized with ketamine and xylazine, then transcardially perfused with 150-200 ml PBS followed by 4% paraformaldehyde. The spinal cord was dissected and maintained for two days in 4% paraformaldehyde. Fixed samples were harvested for subsequent histological processing. Tissue specimens were embedded in paraffin (Merck, Germany) and 5 mm paraffin sections were transversely cut. For immunofluorescence studies, after deparaffinization and antigen retrieval, we rinsed the sections with PBS. Next, the sections were treated with blocking solution and incubated overnight with anti-SSEA-1 at 4˚C. The next day after washing with PBS, the sections were incubated with the secondary antibody, goat anti-mouse (1:1000, Abcam, USA), for 2 hours at room temperature. Sections were washed in PBS, and then nuclei were counterstained with Hoechst for 45 minutes and mounted on glass slides. 

### Real-time reverse transcriptase-polymerase chain reaction (RT-PCR)

We analyzed *Oct4* mRNA levels by isolating total mRNA from spinal cord tissue with Trizol reagent (Invitrogen, USA) according to the manufacturer’s instructions. mRNA was reverse-transcribed with a cDNA synthesis kit (Roche, USA). Detection of Oct4 mRNA levels was performed by real-time RT-PCR using an ABI PRISMs 7000 Sequence Detection System (ABI, USA). The 25 µl reaction mixture contained 12.5 µl SYBR Green PCR Master Mix, 10 ng of cDNA template, and 5ˊ-TGGGGCGGTTTTGAGTAATCT-3ˊ forward and 5ˊCTCTTCTGCTTCAGCAGCTTG-3ˊ reverse primers for *Oct4*. These primers were designed on the basis of areaction with mouse cells. The threshold cycle (Ct) or the cycle number at which the amount of amplified gene of interest reached a fixed threshold was subsequently determined. 

We have calculated the relative relative quantification of *Oct4* mRNA expression according to the comparative Ct method. The relative quantificationvalue of the target, normalized to an endogenous control *β-actin* gene and relative to a calibrator, is expressed as 2^-ΔΔCt^ (fold difference) where ΔCt equals the Ct of the target gene minus the Ct of the endogenous control gene (*β-actin*). Reactions have been performed with appropriate negative controls (template-free) and a uniform amplification of the products was rechecked by an analysis of the melting curves of the amplified products. The melting temperature (Tm) was 57- 60˚C. Gel electrophoresis was performed to confirm the correct size of the amplification and the absence of unspecific bands. 

### Statistical analysis 

Results were expressed as mean ± SD. The statistical significance between the mean values was determined by one-way ANOVA followed by Tukey’s post-test with P<0.05 as the statistically significant criterion. 

## Results

### Isolation and expansion of very small embryoniclike stem cells

**Fig.1:**
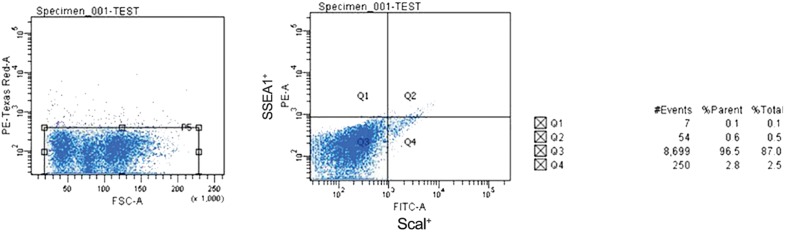
Fluorescent activated cell sorting (FACS) analysis for the isolation of very small embryonic-like (VSELs) stem cells from total bone marrow (BM) cells. Sorting was based on the immunophenotype [Sca1+ and stage specific embryonic antigen (SSEA-1+)] of the VSELs. Events contained the presence of Sca1 and SSEA-1 (gate Q2), which represented the population of VSELs.

We employed multiparameter analysis to isolate VSELs from BM. After flushing the BM cavity, VSELs were isolated using Sca1 and SSEA-1 antibodies and FACS. Reanalysis of sorted cell fractions showed a purity of >95% (Fig .1). 

**Fig.2:**
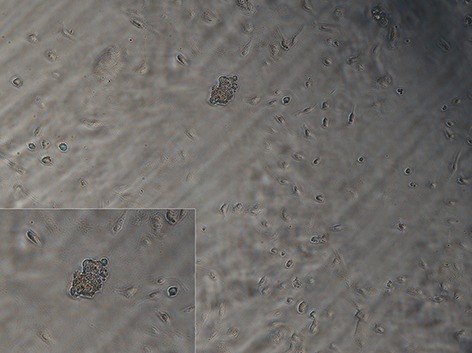
Morphology of very small embryonic-like (VSEL) stem cells derived from 2-4 week-old mice. After 7 days, VSELs began to form sphere-like clusters.

**Fig.3:**
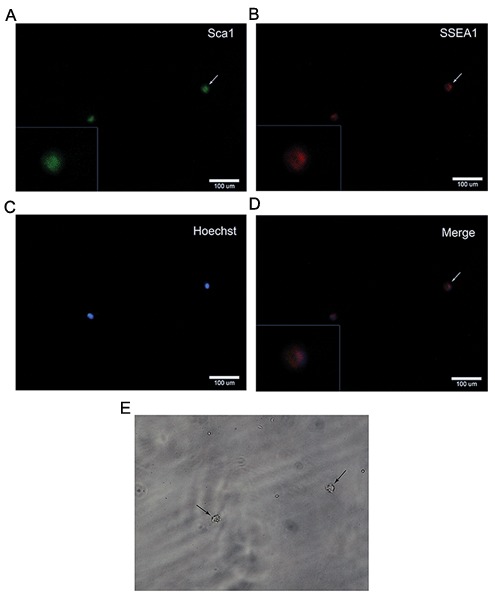
A-D. Characterization of very small embryonic-like (VSEL) stem cell colonies by double staining. VSEL colonies were positive for both Sca and stage specific embryonic antigen (SSEA-1) and E. Light microscopy of single cells showed their very small sizes.

Freshly isolated VSELs were expanded by culturing them on a C2C12 myoblast feeder layer. After 4-7 days, cells began to form sphere-like clusters that consisted of a few hundred cells which resembled embryoid bodies (Fig .2). These colonies were immunopositive for both Sca1 and SSEA-1 which indicated that the colonies were composed mainly of VSELs (Fig .3). 

### Transplantation and survival of very small embryonic-like stem cells into the lesion site

**Fig.4:**
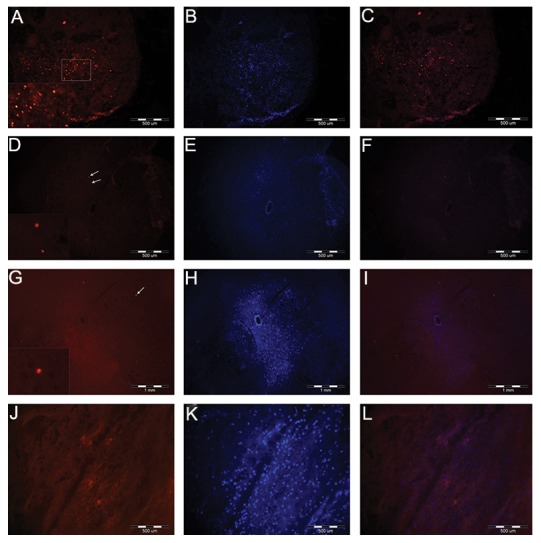
A-C. Cross-section of spinal cord at 4 weeks after very small embryonic-like (VSEL) stem cell transplantation. More labeled VSELs were detected in the dorsal columns of the spinal cords of experimental rats, D-F. Compared with control rats, G-I. Only a few labeled cells were detected in the uninjured site of the experimental rats and J-L. There were no labeled cells within the spinal cords of the sham group. A, D, G, J. Labeled VSELs with 1,1`-dioctadecyl-3,3,3`,3`-tetramethylindocarbocyanine perchlorate (DiI), B, E, H, K. Hoechst positive nuclei, C, F, I and L. Merged.

**Fig.5:**
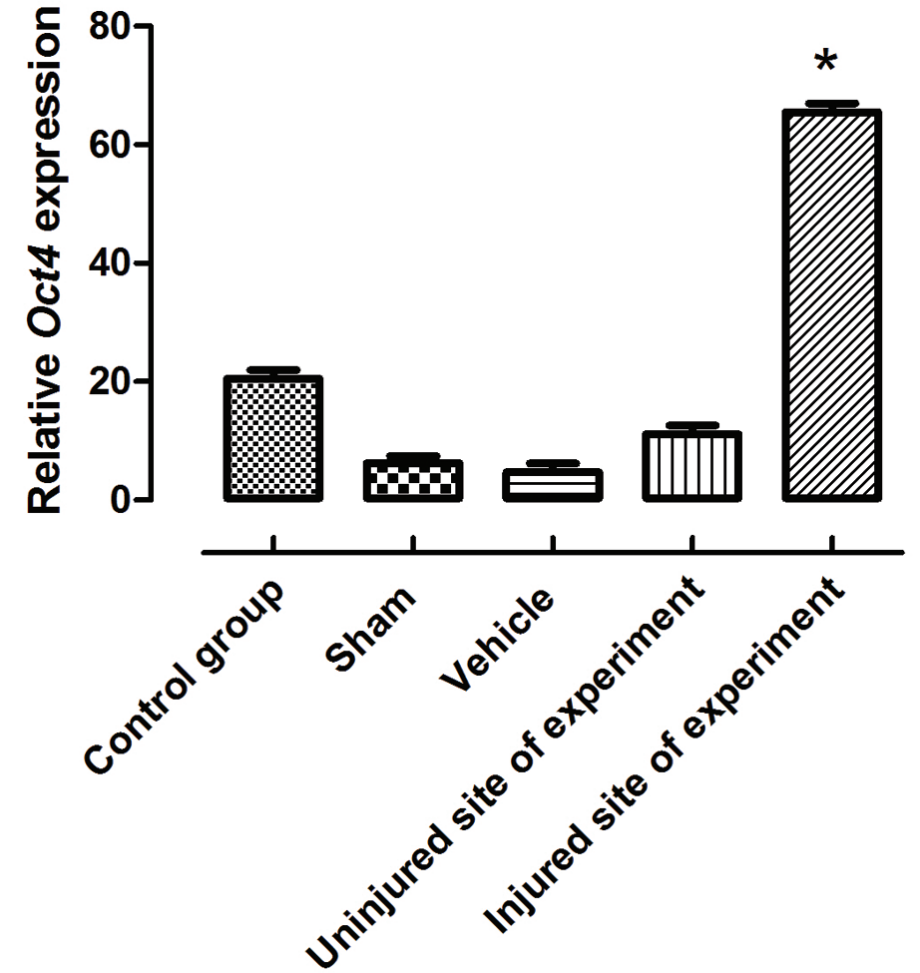
Expression pattern of Oct4 at one month after transplantation as analyzed by real time RT-PCR. The level of Oct4 significantly increased in the injured site of the experimental group. Data show means ± SD, *; P≤0.05 and RT-PCR; Reverse transcriptase polymerase chain reaction.

We determinedthe ability of VSELs to efficiently home to the lesion site. After one month, the lesion site was analyzed in the rats that received VSELs. In these rats, we detected the labeled VSELs at the lesion site (Fig .4). Real time RT-PCR results showed a significantly increased Oct4 expression level (P≤0.05) in the SCI group treated with VSELs (Fig .5). 

**Fig.6:**
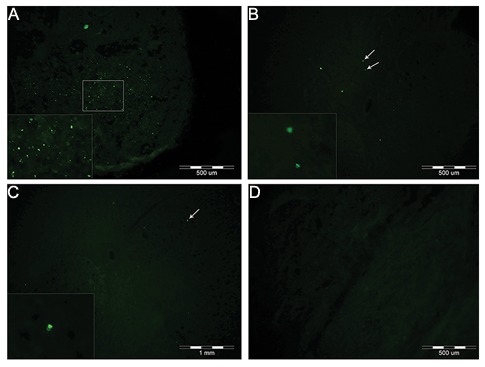
A. Stage specific embryonic antigen (SSEA-1+) cells in the dorsal funiculus of the spinal cord in the experimental, B. Control, C. Uninjured site of experimental and D. Sham groups. Note; There were more SSEA-1+ cells in the experimental group compared to the other groups.

We verified the successful homing of the VSELs by analyzing spinal cord tissues for SSEA-1 which recognizes the VSEL surface marker. SSEA-1 was strongly detected in the group transplanted with VSELs. This result implied that VSELs had migrated into the injured spinal cord via the peripheral circulation and localized into the spinal cord lesion (Fig .6). 

## Discussion

We have transplanted VSELs into the tail veins of rats wherethese cells showed their ability to move toward the injured spinal cord. It is estimated that more than 2.5 million people live with SCI and 130,000 new cases are reported each year. Stem cell therapy is a promising treatment for SCI (15). Kucia et al. (7) have recently discovered a new population of stem cells (VSELs) in BM. A major problem with VSELs is the rare occurrence of these particular cells in BM as low as 0.05% of total BM cells in mice (16). We have used 2-4 week-old mice in this study in order to isolate VSELs. The numbers of VSELs decrease with age and the expression levels of pluripotent markers is higher in VSELs from young animalse (17). 

In this study we enriched VSELs by FACS using SSEA-1 and Sca1 antibodies. Evaluation of the purity of the VSELs preparation demonstrated that this procedure was very effective for VSEL isolation. Other studies evaluated this method when producing VSELs from cord blood as well as PB (8,18). When VSELs culture without any feeder layer or inhibitor, like embryonic stem cells can differentiate spontaneously, thus in this study we used C2C12 feeder layer cells to inhibit their spontaneously differentiation (19). During cultivation, VSELs started colony formation and largely expressed *SSEA-1, Sca1* and *Oct4*. These developmental criteria suggested the proliferation of VSELs into a pluripotent phenotype. 

We have transplanted labeled VSELs through the tail veins of rats. There are several routes for cell transplantation such as: local (directly into the ischemic area), intra-arterial, epidural and intravenous (IV) infusion. Among these, site-directed delivery of cells provides effective engraftment, however it is not always applicable in clinical practice. IV injection is minimally invasive and the majority of injected cells reach their target (20). Previous studies have described the IV route of cell administration and shown this methodto be the most convenient for cell transplantation (21,24). 

In the present study, we established that intravenously administered VSELs have the ability to move towards the lesion site. Another important observation during this study was that the expression level of *Oct4* increased after transplantation of VSELs into the lesion. In parallel, immunohistochemical studies demonstrated that SSEA-1 positive cells localized into the injured spinal cord in the VSEL treated animals. 

Evidence has shown that VSELs mobilize in PB in response to myocardial ischemia (3), stroke (4,10) and skin burn injury (25). The quiescent VSELs can rapidly proliferate and mobilize with increased migration from their resident BM or other tissue to the blood circulation in response to injury (26). A key chemokine involved in this mobilization is SDF-1 (also termed CXCL12) which acts via its major receptor, CXCR4 that expresses on VSELs. These cells respond to the upregulation of SDF-1 within injured organs and migrate into these areas (12). 

Cell therapy for spinal cord repair has generated considerable enthusiasm in recent years and different types of cells have been used for this purpose (15). In view of the ability of VSELs to express neural (*GFAP, Nestin, b-III tubulin, Olig1, Olig2, Sox2*, and *Musashi-1*) stem cell markers in PBborne nucleated cellsthat circulate in stroke patients as well as in a murine model of stroke, we believe that VSELs which colonize in the injured spinal cord may be involved in SCI regeneration. However, the potential use of these cells in spinal cord tissue regeneration requires further study. 

## Conclusion

The present study indicated that transplanted VSELs could migrate via PB and localize in the injured spinal cord. Hence, VSELs might be a therapeutic option for SCI patients. 
